# Role of integrons, plasmids and SXT elements in multidrug resistance of *Vibrio cholerae* and *Providencia vermicola* obtained from a clinical isolate of diarrhea

**DOI:** 10.3389/fmicb.2015.00057

**Published:** 2015-02-17

**Authors:** Neha Rajpara, Braj M. R. N. S. Kutar, Ritam Sinha, Dhrubajyoti Nag, Hemanta Koley, Thandavarayan Ramamurthy, Ashima K. Bhardwaj

**Affiliations:** ^1^Molecular Biology of Diseases, Department of Human Health and Diseases, School of Biological Sciences and Biotechnology, Indian Institute of Advanced Research, GandhinagarIndia; ^2^Division of Bacteriology, National Institute of Cholera and Enteric Diseases, KolkataIndia

**Keywords:** *Providencia vermicola*, *Vibrio cholerae*, plasmid, integron, multidrug resistance, SXT element

## Abstract

The isolates of *Vibrio cholerae* and *Providencia vermicola* obtained from a diarrheal patient were investigated for genetic elements governing their drug resistance phenotypes. Out of 14 antibiotics tested, *V. cholerae* Vc IDH02365 isolate showed resistance to nine antibiotics, while *P. vermicola* Pv NBA2365 was found to be resistant to all the antibiotics except polymyxin B. Though SXT integrase was depicted in both the bacteria, class 1 integron was found to be associated only with Pv NBA2365. Integrons in Pv NBA2365 conferred resistance to β-lactams, aminoglycosides, and trimethoprim. Pv NBA2365 carried two transformable plasmids imparting distinct antibiotic resistance traits to their *Escherichia coli* transformants. In rabbit ileal loop assays, Pv NBA2365 did not show any fluid accumulation (FA) in contrast with Vc IDH02365 that showed high FA. To the best of our knowledge, this is the first report of a highly drug resistant *P. vermicola* and additionally co-existence of multidrug resistant *V. cholerae* and *P. vermicola*. Both the microbes appeared to possess a wide array of mobile genetic elements for a large spectrum of antimicrobial agents, some of which are being used in the treatment of acute diarrhea.

## INTRODUCTION

Diarrhea is one of the infectious diseases that pose a serious threat to humankind. It still remains a common cause of illness and death in developing countries and is caused by bacteria, viruses, or parasites (Navaneethan and Giannella, 2008). Cholera is one of these diarrheal diseases caused by the bacterium *Vibrio cholerae.* More than 200 serogroups of *V. cholerae* are known, out of which only two, O1 and O139 serogroups cause epidemics or outbreaks (Kaper et al., 1995; Faruque et al., 1998). Non-O1/non-O139 serogroups of *V. cholerae* have been associated with sporadic cases of diarrhea and have been implicated as progenitors of new variants of epidemic *V. cholerae* strains (Faruque et al., 2003). There are many reports of mixed infections of *V. cholerae* with other diarrheal pathogens such as *Shigella* spp*., Salmonella* spp.*, Campylobacter* spp*.,* and viruses (Faruque et al., 1994; Enzensberger et al., 2005; Mukherjee et al., 2009). Bacteria belonging to the genus *Providencia* are responsible for human infections such as urinary tract infections, endocarditis, ocular infections, traveler’s diarrhea, and gastroenteritis (Murata et al., 2001; Krake and Tandon, 2004; Yoh et al., 2005; Koreishi et al., 2006). One of the members, *Providencia vermicola,* was first isolated from infective juveniles of the entomopathogenic nematode (Somvanshi et al., 2006). It has been shown to be a symbiont required for successful parasitic life of the nematode in the target insect such as oriental beetle (Young-Keun et al., 2007). Due to its entomopathogenicity, the nematodes carrying *P. vermicola* are used as biopesticides (Young-Keun et al., 2007). The reports pertaining to the drug resistance of *P. vermicola* are non-existent.

Though the diarrheal diseases still remain a scourge, their treatment is becoming more complicated with the increasing problem of multiple drug resistance exhibited by the etiological agents (Sack et al., 2001; WHO, 2014). Emergence of multiple drug resistance causes a serious clinical problem like increase in morbidity and mortality rates (Dalsgaard et al., 2000; Ghosh and Ramamurthy, 2011). All the bacteria including *Vibrio* spp. and *Providencia* spp. have been reported to exhibit multidrug resistance which seriously hampers the containment of infections caused by them (Marchandin et al., 1999; Ghosh and Ramamurthy, 2011). There are several different mechanisms by which bacteria are known to acquire/disseminate drug resistance. Some of the mechanisms are mobile genetic elements such as integrons, transposons and plasmids and numerous inherent mechanisms like mutations in target genes or eﬄux pumps, to name a few (Ghosh and Ramamurthy, 2011; Bhardwaj and Mohanty, 2012; Bhardwaj et al., 2014). Mobile genetic elements play a crucial role in the rapid acquisition and dissemination of drug resistance genes thus manipulating the genome plasticity in accordance with the changing environment (Bhardwaj et al., 2014). Integrons work as platforms that acquire open reading frames by site-specific recombination and convert them into functional form by their expression driven by a resident promoter at the 5′ end of integrons (Stokes and Hall, 1989). SXT element is an integrative and conjugative element (ICE) that acts as a vehicle for translocating many genes including the antibiotic resistance genes (Waldor et al., 1996). This element was first described in *V. cholerae* MO10 isolated from Madras, India in 1992, where it encoded resistance to trimethoprim, sulfamethoxazole, streptomycin, and chloramphenicol (Waldor et al., 1996). ICEs are a type of conjugative transposons that are not capable of autonomous replication and therefore, they integrate and replicate with the chromosomal DNA of the host bacterium (Burrus and Waldor, 2004). Plasmids known as R plasmids (R for resistance), capable of autonomous replication, play an important role in the spread of antibiotic resistance among bacteria (Sack et al., 2001). Earlier work from this laboratory has described the role of some of these genetic elements in determining the multidrug resistance phenotype displayed by the clinical isolates of *V. cholerae* and *Vibrio fluvialis* from Kolkata, India (Rajpara et al., 2009; Mohanty et al., 2012; Singh et al., 2012; Kutar et al., 2013). In addition, inhibition of eﬄux pumps and quorum sensing as alternatives for control of multidrug resistance have also been reviewed (Bhardwaj and Mohanty, 2012; Bhardwaj et al., 2013). Present study describes an interesting case of mixed occurrence of *V. cholerae* and *P. vermicola* in a stab culture from a clinical sample of a diarrheal patient. Results reveal the exquisite contribution/interplay of plasmids, integrons, and SXT elements in imparting the multidrug resistance traits to both the organisms.

## MATERIALS AND METHODS

### BACTERIAL ISOLATES, THEIR IDENTIFICATION, AND DNA PREPARATION

*Vibrio cholerae* (referred to as Vc IDH02365) and *Providencia vermicola* (referred to as Pv NBA2365) were isolated from a stab culture. This stab culture was obtained from the alkaline peptone water (Atlas, 2010) enriched diarrheal sample from a 37 years old male patient with acute watery diarrhea and severe dehydration admitted to the Infectious Diseases Hospital (IDH), Kolkata, India, in 2009. The procedure of obtaining patient sample and his consent was approved by the Institutional Ethical Clearance Committee, National Institute of Cholera and Enteric Diseases (NICED), Kolkata, which conducts an active surveillance of diarrheal diseases at the IDH, Kolkata. All the strains used in the study are described in **Table 1**. For identification of the bacterial isolates, biochemical analysis was performed by the standard protocols (WHO, 1987). 16S rRNA sequencing was performed to confirm the identity of *P. vermicola* using the primers 8F and 1492R (**Table 1**). Genomic and plasmid DNA extraction was performed as described previously (Thungapathra et al., 2002). The study was approved by the Institutional Biosafety Committee (IBSC) of the Indian Institute of Advanced Research, Gandhinagar, and Review Committee on Genetic Manipulation (RCGM) governed by guidelines laid down by the Department of Biotechnology, Government of India.

**Table 1:** List of strains and primers used in the study.

Strains		
**Name of the strain**	**Description**	**Reference**
*Escherichia coli* ATCC25922	Quality control for antibiotic susceptibility test	CLSI (2010)
*E. coli* JM109	Host for electroporation experiments	Yanisch-Perron et al. (1985)
*Vibrio fluvialis* BD146	Positive control for class 1 integron	Rajpara et al. (2009)
*Vibrio cholerae* O139 MO10	Control for SXT element and RAPD analysis	Waldor et al. (1996)
*V. cholerae* O1 El Tor N16961	Control for RAPD analysis	Heidelberg et al. (2000)
*V. cholerae* O1 classical 569B	Reference strain in antibiogram analysis	Kennedy and Richardson (1969)
*V. cholerae* O1 El Tor IDH01526	Control for *V. cholerae*-specific protein OmpW PCR	Kutar et al. (2013)
*V. cholerae* O1 El Tor IDH01572	control for PFGE experiments	Kutar et al. (2013)
*V. cholerae* O1 El Tor IDH01581	Control for PFGE experiments	Kutar et al. (2013)
*V. cholerae* O1 El Tor *Vc IDH02365*	Strains under study	This study
*Providencia vermicola* Pv NBA2365	Strains under study	This study
*P. vermicola* BAB-812	Control for *Providencia-*specific PCR	Gujarat Biodiversity Gene Bank, Gujarat State Biotechnology Mission, Gujarat, India
*Providencia rettgeri* MTCC8099	Control for *Providencia-*specific PCR	Microbial type culture collection, Chandigarh, India

**Primers**		
**Name of primer**	**Primer sequence (5′->3′)**	**Reference**

8F	AGAGTTTGATCCTGGCTCAG	Eden et al. (1991)
1492R	ACGGCTACCTTGTTACGACTT	Eden et al. (1991)
Provi_F	CGCATAATCTCTTAGGAGCAAA	This study
Provi_R	ATGAATCACAAAGTGGTAAGCG	This study
P_Vermi_R	AAGGAGR(A/G)TGATCCAGCCGCAG	This study
OmpW-F	CACCAAGAAGGTGACTTTATTGTG	Nandi et al. (2000)
OmpW-R	GAACTTATAACCACCCGCG	Nandi et al. (2000)
1281	AACGCGCAAC	Akopyanz et al. (1992)
1283	GCGATCCCCA	Akopyanz et al. (1992)
L2	GACGATGCGTGGAGACC	Maguire et al. (2001)
L3	CTTGCTGCTTGGATGCC	Maguire et al. (2001)
InF	GGCATCCAAGCAGCAAGC	Dalsgaard et al. (2000)
InB	AAGCAGACTTGACCTGAT	Dalsgaard et al. (2000)
Qac EΔ1-F	ATCGCAATAGTTGGCGAAGT	Dalsgaard et al. (2000)
Sul1B	GCAAGGCGGAAACCCGCC	Dalsgaard et al. (2000)
SXT-F	TTATCGTTTCGATGGC	Thungapathra et al. (2002)
SXT-R	GCTCTTCTTGTCCGTTC	Thungapathra et al. (2002)

### PULSED FIELD GEL ELECTROPHORESIS (PFGE) AND RANDOM AMPLIFICATION OF POLYMORPHIC DNA (RAPD) ANALYSIS

Pulsed field gel electrophoresis (PFGE) analysis was carried out as described previously (Parsons et al., 2007). For PFGE, 1% Bio-Rad pulsed field certified agarose gel (Bio-Rad Laboratories, Richmond, CA, USA) was prepared in 0.5X TBE and run in CHEF MAPPER (Bio-Rad Laboratories) using autoalgorithm mode (molecular weight range: 100–350 K and a run time of 19 h). The gel was stained with 0.05 mg ml^-1^ ethidium bromide for 30 min and destained with sterile water for 1 h. RAPD analysis was carried out by 1281 and 1283 primers as described earlier (Akopyanz et al., 1992; Chakraborty et al., 2001).

### ANTIMICROBIAL SUSCEPTIBILITY TESTS

The isolates were tested for their susceptibility to ampicillin (10 μg), chloramphenicol (30 μg), co-trimoxazole (1.25 μg trimethoprim/23.75 μg sulfamethoxazole), ciprofloxacin (5 μg), gentamicin (10 μg), streptomycin (10 μg), sulfisoxazole (300 μg), trimethoprim (5 μg), tetracycline (30 μg), neomycin (30 μg), nalidixic acid (30 μg), norfloxacin (10 μg), kanamycin (30 μg), and polymyxin B (300 units) by the disk diffusion method using commercial disks (HiMedia, Mumbai, India) in accordance with the criteria recommended by Clinical and Laboratory Standards Institute (CLSI) standards (CLSI, 2010). When no interpretive criteria for *V. cholerae* were available based on CLSI guidelines, breakpoints for Enterobacteriaceae were applied (CLSI, 2010). For *P. vermicola*, breakpoints for Enterobacteriaceae were applied as it belongs to the same family. *Escherichia coli* ATCC 25922 was used for quality control. Experiments were performed at least two times.

### POLYMERASE CHAIN REACTIONS (PCRs)

All the primers used in this study have been described in **Table 1**. Polymerase chain reaction (PCR) for analysis of SXT elements and class 1 integrons were carried out as described before (Rajpara et al., 2009). Primer pairs L2/L3 (specific for 5′ conserved segment [5′ CS] of class 1 integron), qacEΔ1/Sul1B (specific for 3′ conserved segment [3′ CS] of class 1 integron), In-F/In-B (specific for variable region of class 1 integron) were used for detection and characterization of class 1 integrons using the same conditions excepting that for the primer pair In-F/In-B, annealing was carried out at 63.5^∘^C for 1 min. Reactions were also performed for confirmation of *V. cholerae* isolate using OmpW-specific primer, and confirmation of *P. vermicola* isolate using the species-specific primers Provi_F, Provi_R, and P_Vermi_R. In PCR for *Providencia* sp. (using Provi_F and Provi_R to detect both *P. rettgeri* and *P. vermicola*), annealing was carried out at 62^∘^C for 1 min and polymerization was carried out at 72^∘^C for 1.5 min, while in PCR for *P. vermicola* (using Provi_F and P_Vermi_R to detect only *P. vermicola* and not *P. rettgeri*)*,* annealing was carried out at 68^∘^C for 1 min and polymerization was carried out at 72^∘^C for 1.5 min. PCR reactions were performed using a PTC-225 DNA Engine Tetrad^TM^ Cycler (MJ Research Inc., Waltham, MA, USA). Recombinant *Taq* DNA polymerase (Fermentas International Inc., Burlington, ON, Canada) was used along with appropriate buffers.

### TRANSFORMATION

Transformation of *E. coli* JM109 was carried out by electroporation (Gene Pulser XCell; Bio-Rad Laboratories, Richmond, CA, USA) using 200 ng of total DNA or plasmid DNA from Pv NBA2365 and Vc IDH02365 isolates. Transformants were selected on Luria-Bertani (LB; Lennox, 1955; Atlas, 2010) plates containing ampicillin (25 μg ml^-1^). Electrocompetent cell preparation and electroporation preset protocols used for *E. coli* cells were as per the manufacturer’s instructions.

### DNA SEQUENCING AND SEQUENCE ANALYSIS

DNA segments amplified from integrons and SXT integrases were sequenced. DNA sequencing was performed by Sanger’s chain termination method using DNA sequencer (Applied Biosystems; 3730/3730xl DNA analyzer). For larger segments of DNA, primer walking was carried out and the sequence was assembled. The assembled sequences were analyzed by BLAST search and the sequences were submitted to GenBank. The ORF (Open Reading Frame) Finder tool at the National Center for Biotechnology Information (NCBI) website^1^ was used to predict all the possible ORFs in these sequences.

### RABBIT ILEAL LOOP ASSAY

Rabbit ileal loop experiments were conducted as described earlier (De and Chatterje, 1953; Rajpara et al., 2013). New Zealand White male rabbits (1.5–2 kg) were fasted for 48 h prior to surgery and fed only water ad libitum. Rabbits were anesthetized by intramuscular administration of ketamine (35 mg kg^-1^ of body weight) and xylazine (5 mg kg^-1^). A laparotomy was performed, and the ileum was washed and ligated into discrete loops of approximately 10 cm. Each loop was inoculated with 10^8^ CFU of challenge strain (*V. cholerae* O1 El Tor N16961, Vc IDH02365, Pv NBA2365 and the mixture of Vc IDH02365 and Pv NBA2365) in phosphate-buffered saline (PBS). PBS was used as negative control. The intestine was returned to the peritoneum and the animals were sutured and returned to their cages. After 18 h, rabbits were sacrificed by intravenous injection of pentobarbital (150 mg kg^-1^), and the loops were excised. Fluid volume and loop length were measured, and secretion was recorded as milliliters per centimeter. For rabbit ileal loop assay, the study was approved by the Institutional Ethical Clearance Committee of NICED. All surgery was performed under sodium pentobarbital anesthesia, and all efforts were made to minimize suffering.

### GenBank SUBMISSIONS

The accession numbers for the gene sequences submitted to GenBank from this study were: KC709647, KC709648 for sequences of SXT integrases from Vc IDH02365 and Pv NBA2365 respectively, KC709650, KC709653, KC709649, KC709652 for 5′ CS, 3′ CS, 1.2 kb variable region and 2.8 kb variable region respectively of class 1 integron from Pv NBA2365, and KC709651 for 16SrRNA of Pv NBA2365.

## RESULTS

### CLINICAL ISOLATE CONSISTED OF *V. cholerae* AND *P. vermicola*

**FIGURE 1:**
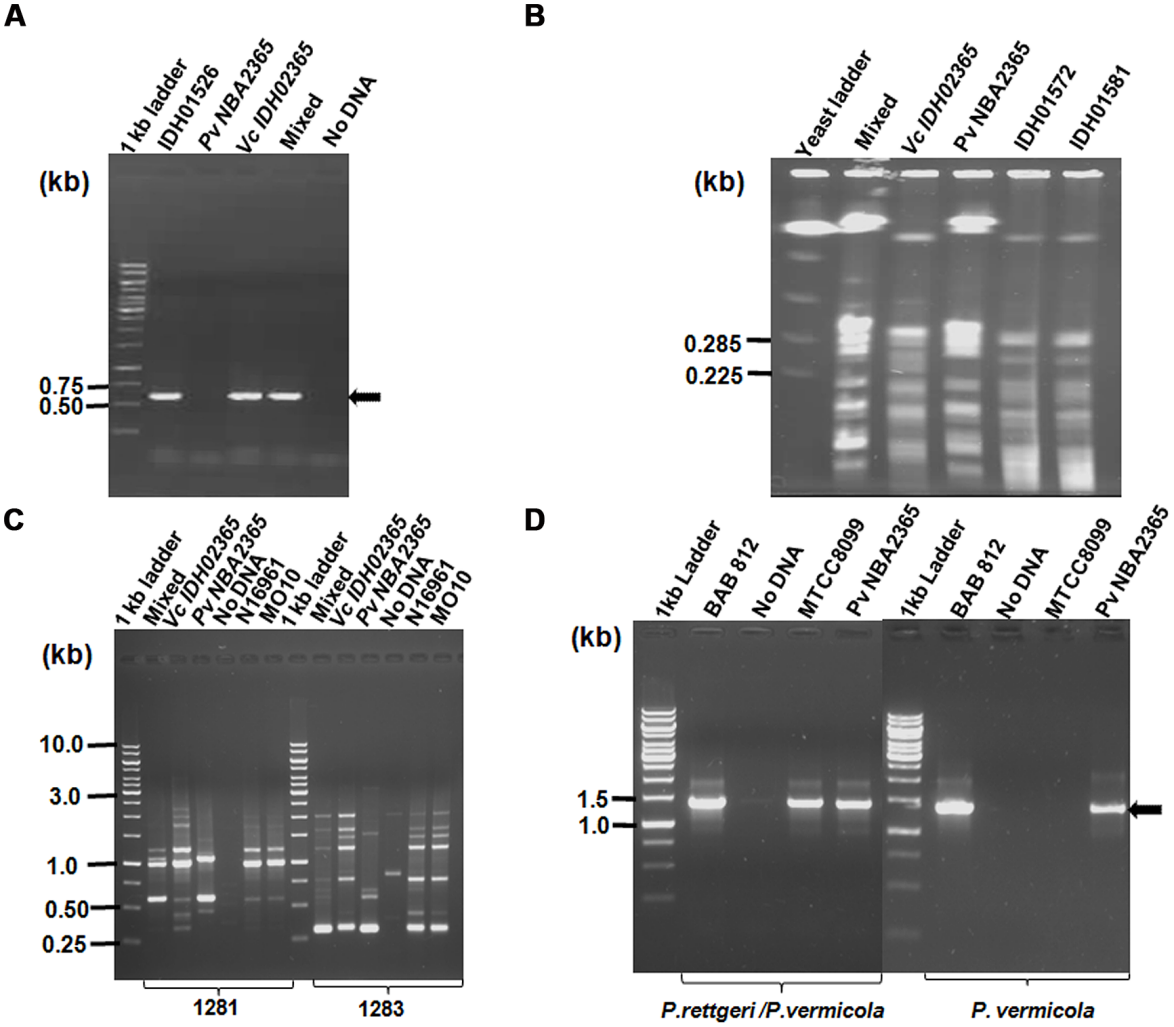
**Identification and differentiation of constituent bacteria of a clinical isolate of diarrhea.** Agarose gel (1%) analysis of **(A)** PCR products for confirming *V. cholerae* species with primer pair OmpW- F/OmpW-R; **(B)** PFGE of DNA from Vc IDH02365 and * P. vermicola* Pv NBA2365 digested with *Sfi*I restriction enzyme; **(C)** RAPD profile of clinical isolates with 1281 and 1283 primers; and **(D)** PCR with species-specific primers for *P. rettgeri/P. vermicola* and only *P. vermicola.* All the DNA samples used as templates are indicated on top of each lane. The clinical isolates of *V. cholerae* IDH01526, IDH01572, IDH01581, N16961, MO10, *P. vermicola* BAB 812, *P. rettgeri* MTCC8099 and mixture of Vc IDH02365 and Pv NBA2365 as obtained from the original stab culture (mixed) were used as controls for various assays. Marker positions in kb have been indicated in the left of each panel. Arrows indicate the position of relevant amplicons for each PCR.

The clinical isolate from diarrhea patient was received in the form of a stab culture from NICED as part of a collection of 119 *V. cholerae* isolates of 2009. It showed the growth of two bacteria with different colony morphologies, i.e., one yielded big sized (4 mm in diameter) mucoid yellowish colonies and the other bacterium yielded small (2 mm in diameter) off white colonies on LB medium. On thiosulfate-citrate-bile salts-sucrose (TCBS) agar (Morris et al., 1979; MacFaddin, 2000), *V. cholerae* grew as sucrose fermenting yellow color colonies, while the other bacteria appeared as bluish green colonies. These bluish green colonies turned yellow when incubated for durations longer than 24 h. Genomic DNA was isolated from both the sucrose fermenting and non-fermenting colonies and analyzed in *V. cholerae*-specific OmpW PCR. An amplicon of expected size (586 bp) was obtained only with DNA from sucrose fermenting colonies confirming its identity as *V. cholerae* whereas the DNA from sucrose-non-fermenting colonies did not yield this amplicon (**Figure 1A**). Distinct band patterns obtained by PFGE and RAPD analysis in these two colonies also corroborated the presence of two different bacteria (**Figures 1B,C**). Both the colonies were subjected to a variety of biochemical tests (**Table 2**). The organism constituting the small sucrose non-fermenting colony was found to be oxidase-negative as compared to *V. cholerae* which was oxidase-positive. Therefore, as evident from many other biochemical tests and growth patterns on various media, this clinical isolate consisted of *V. cholerae* and another organism with biochemical and morphological phenotype different from *V. cholerae* (**Table 2**). In order to establish the identity of the non-vibrio bacteria, results of their biochemical tests were fed into ABIS online (Advanced Bacterial Identification Software^2^). Analysis indicated the possibility of this bacterium to be *Providencia rettgeri* or *P. vermicola*. 16S rRNA sequencing was carried out and the sequence established that this bacterium was *P. vermicola* (GenBank submission **KC709651**). Further confirmation using PCR assay with the primers specific for both the species (yielding a major band of expected size in *P. vermicola* samples BAB812, Pv NBA2365 as well as *P. rettgeri* sample MTCC8099) or primers specific only for *P. vermicola* (yielding a prominent band only in *P. vermicola* samples BAB812 and Pv NBA2365) clearly proved the bacterium to be *P. vermicola* (**Figure 1D**)*.* Hereafter, these bacteria were termed as Vc IDH02365 (for this *V. cholerae*) and Pv NBA2365 (for this *P. vermicola*).

**Table 2:** Biochemical tests and morphological analysis of Vc IDH02365 and Pv NBA2365.

	IDH02365 Mixed*	Vc IDH02365	Pv NBA2365
**Biochemical test**
Indole	–	–	–
TSI Slant	Alkaline	Acidic	Alkaline
Butt	Acidic	Acidic	Acidic
H_ 2_S, gas	–	–	–
LIA	LDE+/LDC-/H_2_S-	LDE-/LDC+/H_2_S-	LDE+/LDC-/H_2_S-
Urease	+	–	+
Citrate utilization	+	–	++
Motility	+	+	+
Mannitol	+	+	+
Glucose	+	+	+
Sucrose	–	+	–
Lactose	–	–	–
Oxidase	–	+	–
Catalase	+++	+	+++
**Morphology on selective and differential media**
TCBS Agar	Yellow colonies	Yellow colonies	Bluish green colonies
Luria-Bertani agar	Big and small colonies merged	Big sized mucoid yellowish colonies	Small off -white colonies
MacConkey agar	Lactose non-fermenting	Lactose non-fermenting	Lactose non-fermenting
HEA	Dark green colonies	No Growth	Dark green colonies
XLD	Dark reddish-brown colonies	No Growth	Dark reddish- brown colonies

*Phenotypes recorded after 18–20 h at 37°C.*

TSI, triple sugar iron agar; LIA, lysine iron agar; TCBS, thiosulfate citrate bile salt sucrose; HEA, Hektoen enteric agar; XLD, xylose lysine desoxycholate agar; LDE, lysine deaminase; LDC, lysine decarboxylase.

*Clinical isolate from diarrheal patient consisting of mixture of two bacteria (Vc IDH02365 and Pv NBA2365) as obtained from the stab culture.

### Vc IDH02365 AND Pv NBA2365 SHOWED DISTINCT ANTIBIOTIC RESISTANCE PROFILES

Vc IDH02365 was resistant to co-trimoxazole, nalidixic acid, polymyxin-B, sulfisoxazole and trimethoprim and showed intermediate resistance to ampicillin, ciprofloxacin, streptomycin, and tetracycline. Pv NBA2365 showed complete/intermediate resistance to all the antibiotics except polymyxin-B (**Table 3**). The resistance of Pv NBA2365 to all the antibiotics used in the study was noteworthy. For comparison, other strains of *P. vermicola* and *V. cholerae* were tested in this study. The results showed distinct resistance profiles for various strains of both the organisms (**Table 3**).

**Table 3:** Antibiotic susceptibility profiles of Pv NBA2365, Vc IDH02365, and other reference strains.

	Pv NBA2365	*P. vermicola* BAB812	*P. vermicola* BAB813	Vc IDH02365	*V. cholerae* O1 El Tor N16961	*V. cholerae* 569B
Resistant	AMP, CIP, CO-TRI, GEN, KAN, NAL, NEO, NOR, SUL, TET, TRI, CHL, STR	CO-TRI, POLY-B, TET, TRI, NEO, SUL	POLY-B, TET, CHL, SUL	CO-TRI, NAL, POLY-B, SUL, TRI, AMP, CIP, STR, TET	AMP, STR, POLY-B, NEO, KAN	AMP

*Drug names have been abbreviated: AMP, ampicillin (10 μg); CHL, chloramphenicol (30 μg); CO-TRI, co-trimoxazole (trimethoprim 1.25 μg/sulfamethoxazole 23.75 μg); CIP, ciprofloxacin (5 μg); GEN, gentamicin (10 μg); STR, streptomycin (10 μg); SUL, sulfafurazole (300 μg); TRI, trimethoprim (5 μg); TET, tetracycline (30 μg); NEO, neomycin (30 μg); NAL, nalidixic acid (30 μg); NOR, norfloxacin (10 μg); KAN, kanamycin (30 μg); POLY-B, polymyxin B (300 units). Intermediate resistance as well as complete resistance were considered to be a resistance trait.*

### PRESENCE OF SXT ELEMENT IN Vc IDH02365 AND Pv NBA2365

**FIGURE 2:**
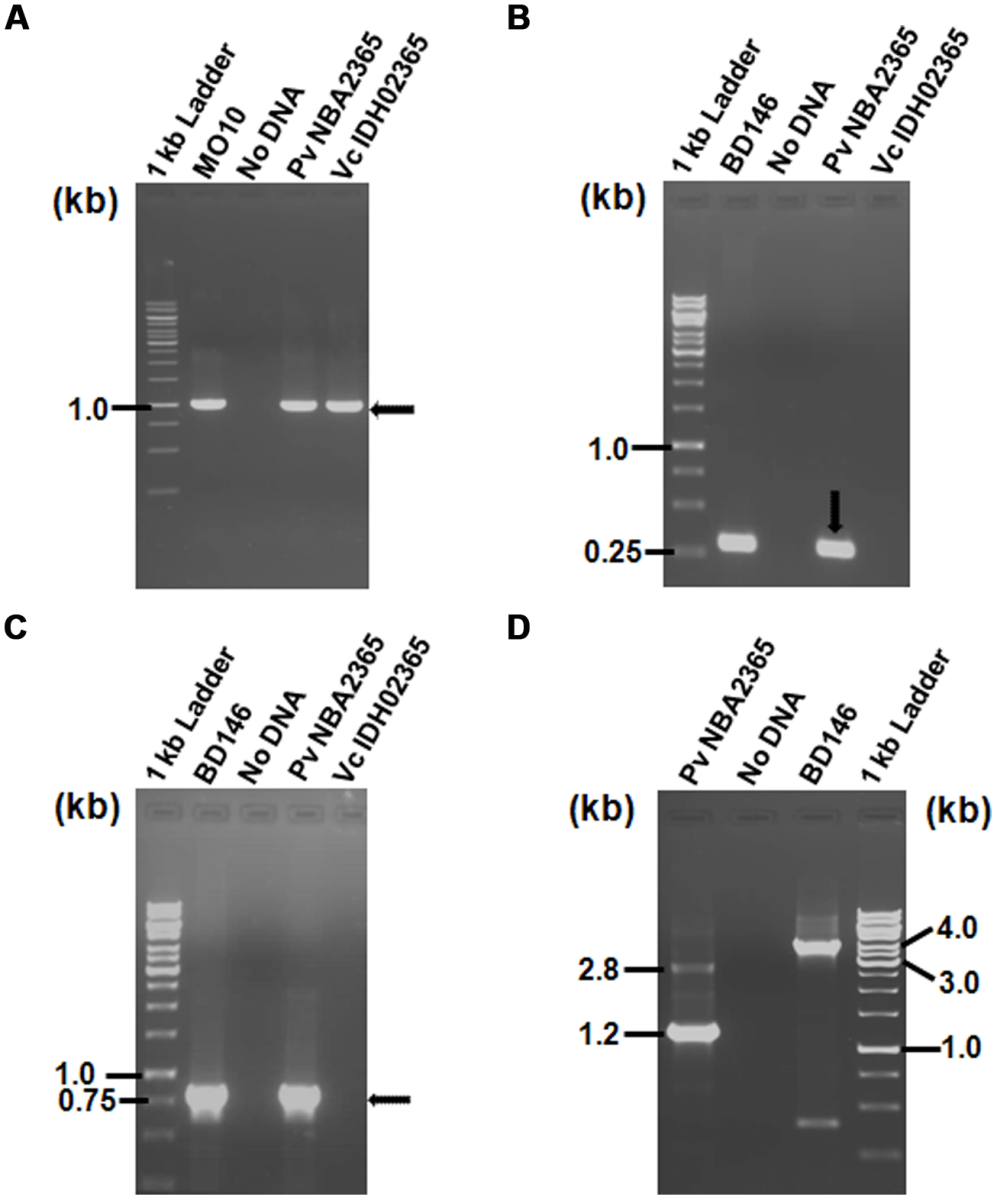
**Agarose gel (1%) of PCR products for analysis of SXT element and class 1 integron showing amplification with **(A)** primer pair SXT-F/SXT-R for detection of SXT integrase; **(B)** primer pair L2/L3 for detection of 5′ CS of class 1 integron; **(C)** primer pair qacEΔ1-F/Sul1-B for amplification of 3′ CS of class 1 integron; **(D)** primer pair In-F/In-B for amplification of variable regions associated with class 1 integrons.** All the DNA samples used as templates are indicated on top of each lane. *V. cholerae* MO10 and *V. fluvialis* BD146 were used as controls for various PCR assays. Marker positions in kb have been indicated in the left of each panel. Arrows indicate the position of relevant amplicons for each PCR.

As the antibiotic resistance profiles of both the bacteria were characteristic of SXT element, PCR experiment was carried out to analyze the presence of this element in these isolates by amplification of SXT integrase. The experiment revealed that both the isolates were positive for SXT integrase (**Figure 2A**) since 1.0 kb amplicon was obtained in Vc IDH02365 as well as Pv NBA2365. Sequences of these amplicons were analyzed by BLAST search at NCBI site. Results showed that SXT integrase from Vc IDH02365 (GenBank submission **KC709647**) had 99% identity with a large number of integrase sequences including the ones from the strains ICE*Vfl*Ind 1 from an Indian isolate of *V. fluvialis* (**GQ463144**), ICE*Vch*Ind5 from Sevagram, India (**GQ463142**), ICE*Vch*Ban5 from Bangladesh (**GQ463140**), and VC1786ICE sequence from Haiti outbreak (**JN648379**). SXT integrase from Pv NBA2365 (GenBank submission **KC709648**) had 98% similarity with integrases from ICE*Pda*Spa1 from *Photobacterium damselae* (**AJ870986**), R997 from *Proteus mirabilis* (**AJ634266**) and ICE*Vch*Ind4 from *V. cholerae* (**GQ463141**). There was 95% similarity between these two SXT integrase nucleotide sequences from Vc IDH02365 and Pv NBA2365.

### Pv NBA2365 CONTAINED TWO CLASS 1 INTEGRONS

**FIGURE 3:**
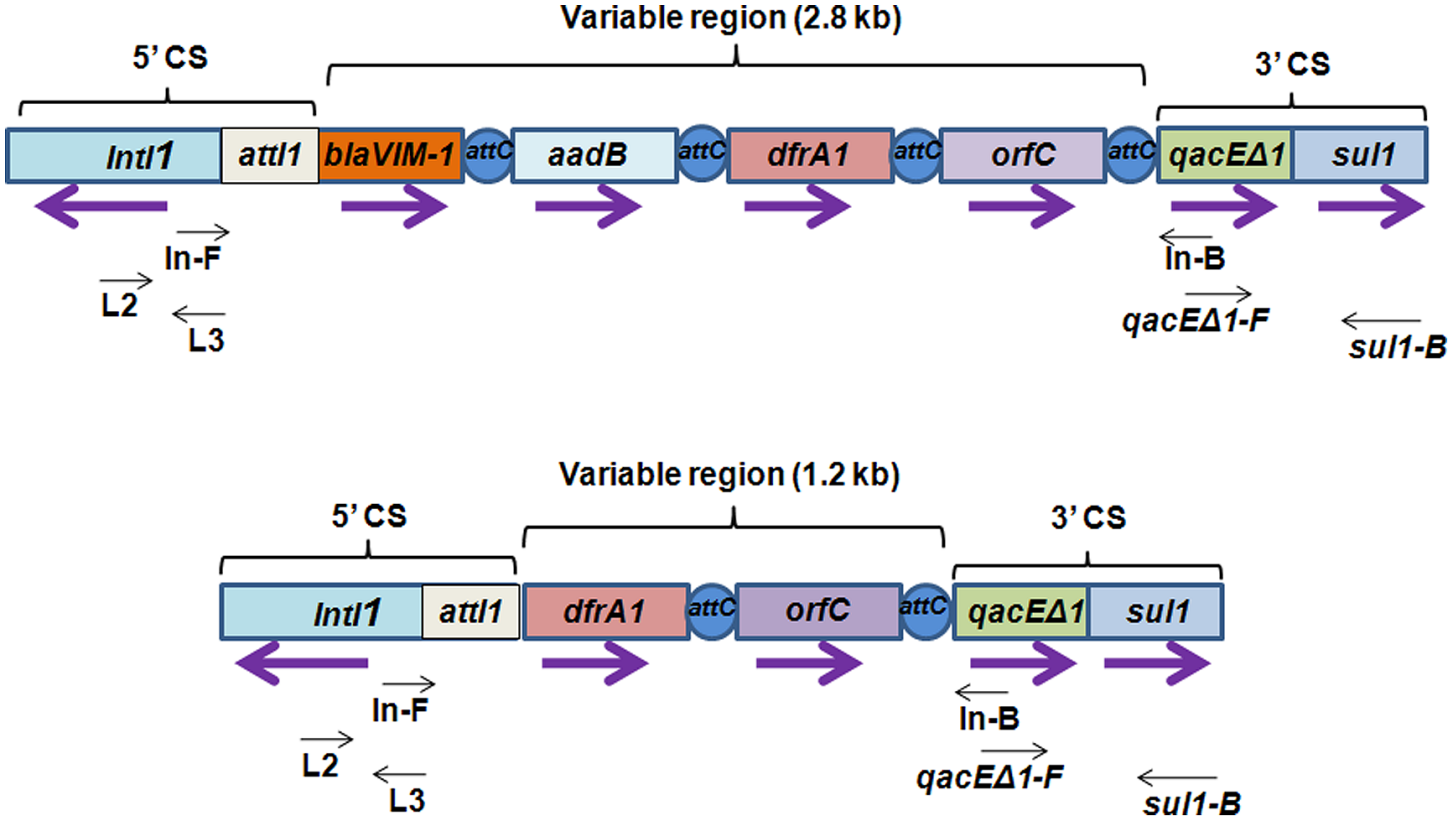
**Schematic representation of class 1 integron containing 2.8 and 1.2 kb variable regions harboring different kinds of gene cassettes.** Integrons consist of a gene *intI1* encoding a site-specific recombinase called “integrase” belonging to tyrosine-recombinase family and a recombination site *attI1* into which exogenous gene cassettes (here *blaVIM-1*, *aadB*, *dfrA1,* and *orfC*) harboring the recombination site *attC* are inserted through site-specific recombination. These exogenous cassettes that vary from one integron to the other, together constitute variable region of the integron. In the 5′ conserved sequences (5′ CS), a promoter located within *intI1* drives transcription of the captured genes. *qacEΔ1* and *sul1* are conserved regions in 3′ conserved sequences (3′ CS) which contribute resistance to ethidium bromide and sulfonamides. Purple arrows indicate the direction of transcription for each gene. Black arrows indicate the position of primers used for detection and analysis of class 1 integrons.

To determine the presence of class 1 integrons as carriers of drug resistance genes, PCR reactions were performed with the primers specific for class 1 integron as described in methods. PCR with primers L2/L3 specific for 5′ CS of class 1 integron yielded an amplicon of around 300 bp in Pv NBA2365 of which 234 bp were sequenced (GenBank submission **KC709650**; **Figure 2B**). Similarly, 3′ CS of about 800 bp (GenBank submission **KC709653**) was obtained with qacEΔ1/Sul1B primer pair in the same organism (**Figure 2C**). These amplicons were not obtained in Vc IDH02365. Moreover, the integron was associated with plasmid(s) in Pv NBA2365 as the bands corresponding to 5′ CS and 3′ CS were obtained with plasmid DNA preparation. Analysis of variable region of integrons amplified using In-F/In-B primers indicated the presence of two major amplicons of 1.2 and 2.8 kb size (**Figure 2D**). The 1.2 kb amplicon (GenBank submission **KC709649**) contained gene cassettes *dfrA1* (encoding group A drug-insensitive dihydrofolate reductase, which is responsible for trimethoprim resistance) and *orfC* (encoding a hypothetical protein). Amplicon with 2.8 kb length (GenBank submission **KC709652**) contained the cassettes *blaVIM-1, aadB, dfrA1,* and * orfC* encoding metallo-β-lactamase, aminoglycoside-2”-adenyl transferase, group A drug-insensitive dihydrofolate reductase and hypothetical protein respectively (**Figure 3**). Therefore, these two integrons present in Pv NBA2365 mediated resistance to ampicillin, trimethoprim, gentamicin, tobramycin, and kanamycin. As was evident from the sequences and the results described above, 1.2 and 2.8 kb cassettes shared some common genes and the identity of 99% in those gene segments.

### PRESENCE OF PLASMIDS IN Pv NBA2365 AND Vc IDH02365

**FIGURE 4:**
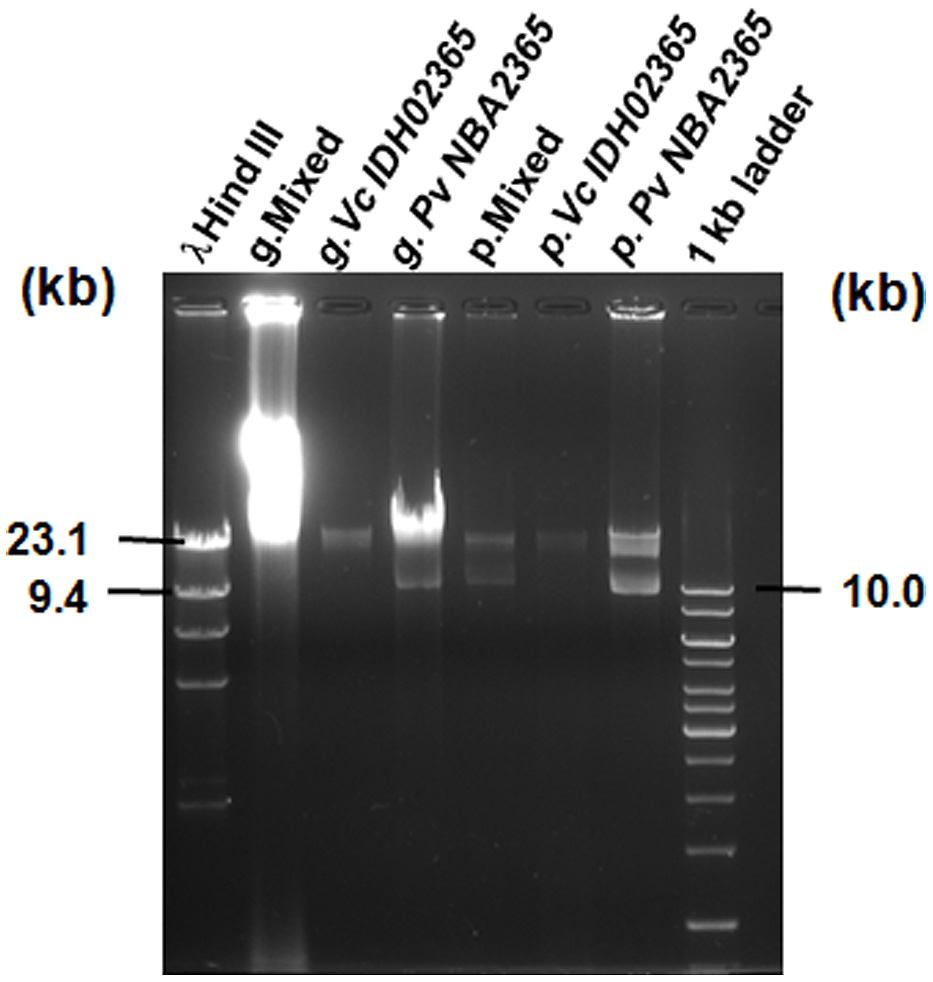
**Agarose gel analysis for genomic DNA and plasmid DNA of Vc IDH02365 and Pv NBA2365.** All the DNA samples used as templates are indicated on top of each lane. Marker positions in kb have been indicated. g. denotes total genomic DNA preparation and p. denotes plasmid DNA preparation.

Agarose gel analysis of total genomic and plasmid DNA from Pv NBA2365 and Vc IDH02365 indicated the presence of multiple plasmid bands in the range of 9.0–23.0 kb (**Figure 4**). To determine the transferability of these plasmids, transformation experiments were carried out and ampicillin selection was used. Conjugation could not be performed as PvNBA2365 was resistant to all the drugs that were tested and therefore, screening of the exconjugants was a problem. Transformants were obtained with the DNA preparations from these bacterial isolates confirming the presence of transferable plasmids in both the bacteria. These transformants were analyzed by agarose gel electrophoresis of their plasmid DNA, antibiotic susceptibility profiles and for integron analysis. Two types of transformants were obtained. First type was negative for integrase of class 1 integron and showed resistance to ampicillin, kanamycin, nalidixic acid, and neomycin (**Table 4**). These transformants were obtained with DNA from both the bacteria. The second type of transformants positive for this integrase, were obtained only with DNA from Pv NBA2365 and displayed resistance to ampicillin, co-trimoxazole, nalidixic acid, kanamycin, sulfisoxazole, and trimethoprim (**Table 4**). This indicated that there were atleast two plasmids associated with Pv NBA2365.

**Table 4:** Antibiotic susceptibility patterns of Vc IDH02365, Pv NBA2365 and their respective *E. coli* JM109 transformants.

Strains	Vc IDH02365	Pv NBA2365
Parent	CO-TRI, NAL, POLY-B, SUL, TRI, AMP, CIP, STR, TET	AMP, CIP, CO-TRI, GEN, NAL, NOR, SUL, TET, TRI, KAN, CHL, NEO, STR
Integrase-positive transformants	NO TRANSFORMANTS	AMP, CO-TRI, NAL, SUL,TRI, KAN
Integrase-negative transformants	AMP, KAN, NAL, NEO	AMP, KAN, NAL, NEO

*Drug names have been abbreviated: AMP, ampicillin (10 μg); CHL, chloramphenicol (30 μg); CO-TRI, co-trimoxazole (trimethoprim 1.25 μg/sulfamethoxazole 23.75 μg); CIP, ciprofloxacin (5 μg); GEN, gentamicin (10 μg); STR, streptomycin (10 μg); SUL, sulfafurazole (300 μg); TRI, trimethoprim (5 μg); TET, tetracycline (30 μg); NEO, neomycin (30 μg); NAL, nalidixic acid (30 μg); NOR, norfloxacin (10 μg); KAN, kanamycin (30 μg); POLY-B, polymyxin B (300 units). Two transformants from each kind of DNA sample were tested for their antibiograms. The experiment was done in duplicates. Intermediate resistance as well as complete resistance were considered to be a resistance trait. *E. coli* JM109 was resistant to nalidixic acid.*

### Vc IDH02365 WAS HIGHLY ENTEROTOXIC IN A RABBIT ILEAL LOOP ASSAY AS COMPARED TO Pv NBA2365

**FIGURE 5:**
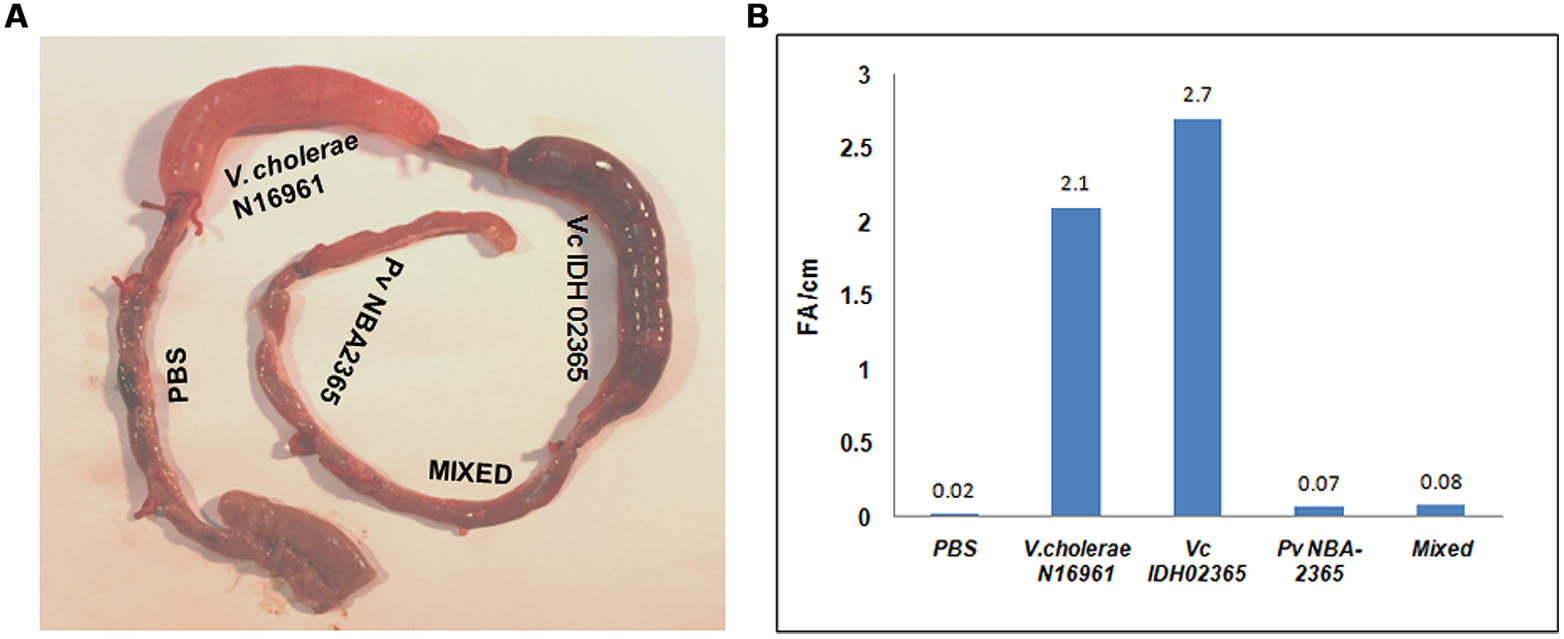
**Rabbit ileal loop assay to assess entrotoxigenic activity.** Pictorial view of rabbit ileal loop of different clinical strains **(A)**. Analysis of fluid accumulation of different clinical strains **(B)**. Rabbit ileal loops were inoculated with 10^8^ CFU of each strain *V. cholerae* N16961 (O1 El Tor, clinical), Vc IDH02365 (O1 El Tor, clinical), Pv NBA2365 in PBS and incubated for 18 h. Results are expressed as fluid accumulation (FA; in milliliters) per loop length (in centimeters). PBS and the mixture of Pv NBA2365 and Vc IDH02365 were taken as controls in this assay.

Vc IDH02365 and Pv NBA2365 were tested in rabbit ileal loop assays to assess their fluid accumulation (FA) potential (**Figure 5**). PBS, the mixture of Vc IDH02365 and Pv NBA2365, and *V. cholerae* O1 El Tor N16961 were taken as controls in this assay. Results revealed that the FA was the highest for Vc IDH02365 (2.7 ml cm^-1^) which was comparable to that of the epidemic strain *V. cholerae* O1 El Tor N16961 (2.1 ml cm^-1^). Pv NBA2365 showed the accumulation of 0.07 ml cm^-1^. The controls of PBS and the mixture of isolates of Pv NBA2365 and Vc IDH02365 showed the FA values of 0.02 and 0.08 ml cm^-1^ respectively.

## DISCUSSION

Bacteria show remarkable adaptability for their survival under stressful conditions (Lee et al., 2010). They evolve rapidly not only by mutations and rapid multiplication, but also by transfer of DNA, which can result in strains with beneficial mutations from more than one parent. Another way is communication/quorum sensing by autoinducers for their mutual survival and fitness (Macleod and Stickler, 2007; Lee et al., 2010). In addition, mobile genetic elements play crucial role in adaptation and evolution through horizontal gene transfer which involves panoply of drug resistance genes and many other genes required to adapt to newer situations (Bhardwaj and Vinothkumar, 2015). In this study, results have been presented for a diarrheal sample containing *V. cholerae* Vc IDH02365 and *P. vermicola* Pv NBA2365. Presence of Pv NBA2365 with Vc IDH02365 could actually be derived from the clinical sample of diarrhea itself (co-infection) or could have been derived as a contamination of the stab culture (contaminant). The possible contamination of the stab could be ruled out as no other strain in the population of 119 strains of *V. cholerae* of 2009 (from where Vc IDH02365 was obtained) harbored *P. vermicola.*

From the data obtained, it appeared that both the organisms utilized a diverse array of genetic elements to manage their survival under pressure from various classes of antibiotics. The high degree of drug resistance harbored by Pv NBA2365 could be of special concern as the organism was found equipped with all possible mobile genetic elements; multiple plasmids, multiple integrons, and SXT element. This clearly reflects the rapid evolution of these organisms through acquisition and dissemination of antibiotic resistance traits in the environment.

Analysis of the antibiotic susceptibility patterns revealed that Vc IDH02365 was resistant to nine and Pv NBA2365 was resistant to thirteen out of the 14 antibiotics used in the study. In order to unravel the molecular mechanisms responsible for the acquisition/dissemination of their drug resistance phenotypes, a study was initiated to understand the contribution of various mobile genetic elements such as SXT element, integrons, and plasmids. Analysis of integrase for SXT element revealed that this element was present in both the organisms. These integrases were 95% identical at nucleotide level and 99% identical at amino acid level. *P. rettgeri* from South Africa was reported to carry an SXT-related IncJ element called R391 in 1967 (Coetzee et al., 1972) and it was hypothesized that *Providencia* spp. could be a primary source of SXT element (Burrus et al., 2006). SXT and R391 integrase genes have been shown to be nearly identical (Hochhut et al., 2001). Comparison of the complete genome sequences of SXT and R391 has also proved the close relationship between these two ICE elements (Beaber et al., 2002). Therefore, though in this study, the complete sequences were not determined for SXT elements from Pv NBA2365 and Vc IDH02365, our findings from the nearly identical nucleotide sequences of SXT integrase genes from these organisms corroborated the earlier findings made by other groups (Hochhut et al., 2001; Beaber et al., 2002).

Integrons were only harbored by Pv NBA2365 and conferred resistance to ampicillin, trimethoprim, gentamicin, tobramycin, and kanamycin. The 1.2 kb variable region of class 1 integron was 99% identical (100% query coverage) to the inetgron sequences from many *Salmonella enterica* sp. (AY963803.6, EF547513.1, DQ641477.1), *V. cholerae* non-O1/non-O139 (AB219453.1, AB219451.1), *V. cholerae* O139 (AB219237.1), *P. mirabilis* (JX089581.1). The 2.8 kb variable region of integron was 99% identical (66% query coverage) to a sequence from *P. mirabilis* (KF501390.1, HQ888851.1, EU259884.2). Electroporation experiments revealed two types of transformants (class 1 integron-positive and class 1 integron-negative) from the Pv NBA2365 while one kind of transformant (class 1 integron-negative) was obtained from Vc IDH02365. Antibiogram analysis revealed common resistance profiles of ampicillin, kanamycin, nalidixic acid, neomycin in Vc IDH02365 transformant and integron-negative transformant of Pv NBA2365*.* Though it still remains to be proved that these organisms shared the same plasmid by virtue of the drug resistance phenotype they conferred upon their respective transformants, there is a possibility that this plasmid was shared between them through horizontal gene transfer.

Therefore, the two organisms utilized all possible mobile genetic elements (SXT element, integrons, and plasmids) to cope with the antibiotic pressure. Though the possibility of other genetic factors in determining antibiotic resistance traits could not be ruled out, a lot more research needs to be carried out to define the complete arsenal that these organisms possessed. In the present study, though Pv NBA2365 did not show pathogenicity as assessed by its FA potential in a rabbit ileal loop assay, it could nevertheless act as a reservoir of multidrug resistance genes. A very recent report has shown the presence of a small plasmid pVERM in *P. vermicola* and hypothesized that this plasmid could have harbored *qnrD* allele for quinolone resistance and transferred it to other bacteria during evolution (Guillard et al., 2014). Hence, this is the first report of a highly drug resistant *P. vermicola* Pv NBA2365 where various genetic elements governing the drug resistance profile of this organism have been unraveled.

## AUTHOR CONTRIBUTIONS

Conceived and designed the experiments: Ashima K. Bhardwaj and Hemanta Koley. Performed the experiments: Neha Rajpara, Braj M. R. N. S. Kutar, Dhrubajyoti Nag, Ritam Sinha, Hemanta Koley. Analyzed the data: Ashima K. Bhardwaj, Thandavarayan Ramamurthy, Hemanta Koley, Neha Rajpara, Braj M. R. N. S. Kutar. Contributed reagents/materials/analysis tools: Ashima K. Bhardwaj, Thandavarayan Ramamurthy, Hemanta Koley. Wrote the paper: Ashima K. Bhardwaj, Thandavarayan Ramamurthy, Braj M. R. N. S. Kutar, Neha Rajpara.

## Conflict of Interest Statement

The authors declare that the research was conducted in the absence of any commercial or financial relationships that could be construed as a potential conflict of interest.

## Footnotes

1

2 http://www.tgw1916.net/bacteria_logare.html
